# Molecular characterization and phylogenetic analysis of fowl adenovirus serotype-4 from Guangdong Province, China

**DOI:** 10.14202/vetworld.2020.981-986

**Published:** 2020-05-24

**Authors:** Fu Yuming, Yuan Sheng, Deng Wenyu, Chi Shihong, Li Wenfeng, Huang Wenjing, Li Xiaowen, Saeed El-Ashram, Kun Mei, Guo Jinyue, Zhang Xuelian, Li Zhili, Huang Shujian

**Affiliations:** 1 Department of Microbiology, College of Life Science and Engineering, Foshan University, Foshan 528231, Guangdong Province, China; 2 Guangdong Women and Children Hospital, Guangdong Province, China; 3 Department of Zoology, Faculty of Science, Kafrelsheikh University, Kafr el-Sheikh 33516, Egypt

**Keywords:** Angara disease, fowl adenovirus-4, phylogenetic analysis

## Abstract

**Aim:**

Our aim in this study was to isolate potentially novel strains of fowl adenovirus serotype-4 (FAdV-4) that is currently circulating in broiler chicken flocks in Guangdong Province, China, and to compare nucleotide and amino acid (AA) sequences of their respective *hexon* genes.

**Materials and Methods:**

The experiment was carried out on poultry farms experiencing outbreaks of FAdV-4-associated hydropericardium syndrome (HPS). Tissue samples from the hearts and livers of deceased chickens were screened for FAdV-4 infection using *hexon* gene-specific polymerase chain reaction (PCR).

**Results:**

New virus isolates were used to infect 7-day-old chicks, which went onto reproduce typical HPS signs. The hypervariable region of the FAdV-4 *hexon* gene was PCR-amplified and sequenced. The *hexon* nucleotide and deduced AA sequence identities were 99.8-99.9% and 99.5-99.8%, respectively, among the four novel isolates. In addition, the new isolates were 97-100% and 96.4-99.9% identical to the nucleotide and deduced AA sequences, respectively, of FAdV-4 *hexon* genes available in the National Center for Biotechnology Information GenBank database. Phylogenetic analyses, based on the *hexon* gene sequence, revealed that the new isolates, clustered with FAdV-C; the FAdV-A, FAdV-B, FAdV-D, and FAdV-E viruses, were more distantly related.

**Conclusion:**

New FAdV-4 isolates from Guangdong Province are similar to those identified in other regions of the world. This information provides critical insight into HPS epidemiology and provides a perspective for monitoring outbreaks and developing strategies for disease prevention.

## Introduction

Adenoviruses comprise a large group of DNA viruses that infect humans, animals, and birds [1]. Fowl adenoviruses (FAdVs) are members of the genus *Aviadenovirus* of the family Adenoviridae. Aviadenoviruses are associated with a variety of diseases, including inclusion body hepatitis (IBH), hydropericardium syndrome (HPS), gizzard erosions, proventriculitis, and tenosynovitis [2]. Aviadenoviruses have been subdivided into five species with 12 serotypes based on their molecular structures and cross-neutralization test results, respectively [3]. There are currently five known FAdV species, including FAdV A (FAdV serotype 1), FAdV B (FAdV serotype 5), FAdV C (FAdV serotypes 4 and 10), FAdV D (FAdV serotypes 2, 3, 9, and 11), and FAdV E (FAdV serotypes 6, 7, 8a, and 8b) [4]. IBH and HPS have been reported by poultry farms from various parts of the world, including within China. FAdV serotype-4 (FAdV-4) has been identified as the causative agent of HPS [5], also known as Angara disease [6].

Clinical HPS cases have been reported since 2015 in poultry farms in China, including those in Shandong, Hubei, Jiangsu, Anhui, Jiangxi, and Henan Provinces; this represents an enormous economic loss for the domestic poultry industry [7]. There has been little to no molecular characterization or phylogenetic analyses of FAdV strains currently circulating in broiler chicken flocks located in Guangdong Province.

The aim of the study was to isolate FAdV-4 strains from chickens in Guangdong Province that were clinically diagnosed with HPS and to investigate the similarities and differences among the nucleotide sequences of their respective *hexon* genes. A more complete understanding of specific FAdV-4 sequences will provide epidemiological information that may lead to improve disease prevention strategies through effective vaccination.

## Materials and Methods

### Ethical approval

All experiments were conducted according to the ethical standards and protocols approved by the Committee of Animal Experimentation of College of Life Science and Engineering, Foshan University, Guangdong, China (permission number 2016- FOSU-CLSE21).

### Collection of samples

Four tissue samples, including hearts and livers of dead chickens with suspected HPS (Angara disease), were collected from five poultry farms in Guangdong Province, China. Signs of disease included pericardial effusion, epicardial fat deposits, and bleeding. The livers were enlarged and fragile with discrete petechial hemorrhages. The samples were stored at −20°C until use.

### Virus isolation

The tissue samples (0.2 mg) were pulverized; the resulting material was suspended in saline and used to inoculate 9-day-old embryonated chicken eggs (ECEs). Normal saline, without liver tissue, was used as a negative control. The ECEs were examined every 12 h after inoculation; the dead embryos were harvested and examined for pathological changes. Allantoic fluids were collected for repeated inoculation of chicken embryos in order to amplify virus [7].

### Hemagglutination (HA) and dipstick tests

To examine virus-mediated HA reactions, allantoic fluids from chick embryos from eggs that were inoculated with liver tissue were added to chicken, rat, guinea pig, and sheep red blood cells [8]. A nucleic acid dipstick test was used to detect avian influenza (AI) and avian leukosis virus (ALV) [9].

### Genomic DNA extraction and hexon gene amplification

Allantoic fluid samples were subjected to centrifugation at 3000 rpm for 10 min; viral DNA was isolated directly from this material using a Viral RNA/DNA Purification kit (Thermo Fisher Scientific China Co Ltd.), according to the manufacturer’s instructions and details noted in El-Ashram *et al*. [10]. A 1008-bp fragment of the *hexon* gene (GenBank No. EU931692.1) was amplified by polymerase chain reaction (PCR) to identify samples. The primers designed for this purpose are listed in Table-1. The PCR reaction included 2× Easy Taq PCR SuperMix, 10 pmol of each primer, and 2 mL of DNA template and nuclease free water to 25 mL. The following PCR conditions were used: Initial denaturation at 94°C for 4 min, 30 cycles of denaturation at 94°C for 30 s, annealing at 66°C (for fragment A) or 57°C (for fragment B) for 30 s, extension at 72°C for 60 s, and a final extension at 72°C for 10 min.

**Table-1:** Sequences of primers employed in the conventional PCR.

Target genes	Primer expected product	bp	Temp
Hexon gene of FAdV-4	F: CCTCCAACAG TTCATTT	1008	47°C
	R: TCTTCGTAAC CGTCATT		

FAdV-4=Fowl adenovirus species C serotype-4, PCR=Polymerase chain reaction

### DNA cloning and sequencing

PCR amplicons were subjected to agarose gel electrophoresis, excised from the gel, purified using an Agarose Gel DNA Purification Kit (TaKaRa, Japan), and cloned into the pMD18-T vector according to the manufacturer’s instructions (TaKaRa, Japan). The recombinant plasmid was transformed into DH5a competent E. coli cells. Plasmid DNA was purified using the E.Z.N.A.® Plasmid Mini Kit I (Omega Bio-Tek, USA) and quantified by spectrophotometric analysis. Recombinant plasmids of fragment A or fragment B were sequenced by Shanghai Sangon Bioengineering Ltd., China with primers included in Table-1. Detailed information, including the GenBank accession numbers of all sequences of the four novel strains, is available in Table-2. ClustalX software (version 1.83, Desmond G. Higgins, Paul Sharp, Trinity College Dublin, Ireland) was used to construct multiple alignments. Phylogenetic trees were constructed usingMEGAsoftware version 5.1 (https://mega.software.informer.com/5.1/), by the neighbor-joining analysis using the Maximum Composite Likelihood algorithm with 1000 bootstrap replicates. The datasets used for phylogenetic datasets included four novel FAdV-4 isolates from this study and 25 reference strains retrieved from GenBank database (Table-3).

**Table-2:** GenBank accession numbers for all sequences of four strains.

S. No.	Date	Host	Age	Strain name	Deposited in GenBank with accession numbers
1	May 17, 2016	Chicken	~20-30 days	CH/GDBL-S1/2016	MK875246
2	February 25, 2017	Chicken	3 days	CH/GDHZ-S2/2017	MK875247
3	February 14, 2017	Chicken	100 days	CH/GDJM-S6/2017	MK875248
4	November 14, 2017	Chicken	60 days	CH/GDHZ-S7/2017	MK875249

**Table-3:** Details of fowl aviadenovirus strains and isolates employed for sequence alignment and phylogenetic analysis.

S. No.	Collection date	Name of strains	Geographic origin	GenBank accession no. *hexon* gene
1	January-1996	VR-432	Phelps	NC_001720.1
2	November-2000	JM1/1	Japan: Kagoshima	MF168407.1
3	November-1995	CELO	Russia	Z67970.1
4	July-2003	Fowl aviadenovirus A	UK	AC_000014.1
5	Early 1970s	340	Ireland	NC_021221.1
6	Early 1970s	340	Ireland	KC493646.1
7	September-2015	C-2B	USA	KT717889.1
8	July-2004	ON1	Canada	NC_015323.1
9	September-2015	ZK	China	KU647689.1
10	October-2015	HN/151029	China	KX090424.1
11	September-2015	SDXT2-15	China	KU877432.1
12	February-2015	HB1502	China	KX421401.2
13	May-2002	Fowl aviadenovirus 4	Indian	AJ459805.1
14	March-2010	Kr-Gunwi	South Korea	HQ709227.1
15	1995	MX-SHP95	Mexico	KP295475.1
16	October-2009	Fowl aviadenovirus C	Russia	KJ207054.1
17	January-2014	FAdV/PA/Layer/27614/13	USA	KU175344.1
18	August-1998	A-2A	Canada	NC_000899.1
19	July-2015	LN/1507	China	KU497449.1
20	January-1995	MX95-S11	Mexico	KU746335.1
21	July-2003	Fowl aviadenovirus D	UK	AC_000013.1
22	1950s/1960s	YR36	Japan	KT862809.1
23	December-2009	764	Canada	JN112373.1
24	November-2015	HLJ/151129	China	KX077988.1
25	2004	UPM04217	Malaysia	KU517714.1

### Macroscopic examination and molecular identification of FAdV-4 isolates

The pathogenicity of the newly isolated FAdV-4 strains was evaluated in 7-day-old specific-pathogen-free (SPF) chickens through intramuscular inoculation of 0.2 mL allantoic fluid from each infected, 9-day-old ECE. Negative controls included 7-day-old SPF chickens that were inoculated intramuscularly with phosphate-buffered saline. Heart and liver samples were collected from deceased birds for further evaluation and virus identification.

## Results

### Macroscopic examination of naturally-infected broiler chickens

**Figure-1:**
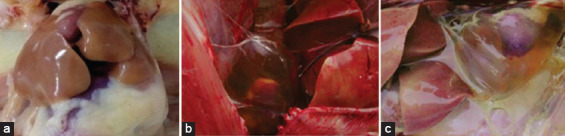
Prominent pathological changes in the fowl adenovirus species C serotype-4-infected chickens (b and c) compared to the uninfected controls (a).

Broiler chickens infected with FAdV-4 showed signs of morbidity, including loss of appetite and lethargy, associated with loose, yellow-green-colored stools. The most common gross lesion at necropsy was gelatinous material accumulating in the pericardial cavity. Other findings included swollen livers with petechial hemorrhages. No gross lesions were detected in uninfected chickens (Figure-1).

### Infection of colon epithelial cells (CECs) with FAdV-4

**Figure-2:**
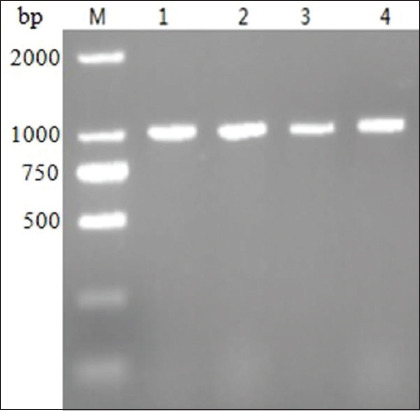
Amplification result of the partial *hexon* gene. M, 2000 bp DNA Marker, lanes (CH/GDBL-S1/2016, CH/GDHZ-S2/2017, CH/GDJM-S6/2017, CH/GDHZ-S7/2017, respectively).

CECs were infected with pulverized liver materials; allantoic fluid was collected; and virus infection was evaluated. No red blood cell aggregation was detected through a HA test. A nucleic acid test strip assay was negative for AI and ALV. PCR amplification with FAdV-4 *hexon* gene-specific primers generated a band of approximately 1008 bp (Figure-2).

### Macroscopic examination of experimentally-infected chicks and molecular identification of FAdV-4 isolates

**Figure-3:**
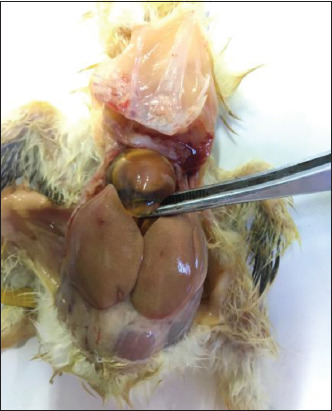
Gross pathology of fowl adenovirus species C serotype-4-infected chicks at necropsy.

The gross changes, identified on necropsy of the experimentally-infected chicks, were consistent with those observed in response to natural infection. The lesions were typical for HPS and included gelatinous material accumulations in the pericardial cavity (Figure-3). No deaths were recorded among the chickens in the uninfected control group. We used the primers listed in Table-2 to amplify a fragment of the *hexon* gene of FAdV-4 virus; liver and heart tissue samples were FAdV-positive by PCR; no amplification was detected in tissues from the uninfected controls.

### Sequence alignment and phylogenetic analysis

We identified four novel isolates of FAdV-4 from infected broiler chickens. The *hexon* genes of each strain include 2814 nucleotides that encode 937 amino acids (AAs). The highly variable regions, based on those identified in the reference SDXT2 (GenBank KU877432.1) strain, included nucleotides 90~200, 988~1233, 2017~2067, and 2650~2679, encoding AAs 330~411, 663~667, 673~689, and 887~893. Among the regions of sequence divergence, we note nine specific AA changes, including A196R, A197G, Y989N, N1042D, D1231N, T2063I, L2066P, M2659V, and L2678P (Table-4).

**Table-4:** Amino acid point mutations of the Chinese strains compared to SDXT2 (KU877432.1).

Strains	Positions of amino acid point mutations							

	66	330	348	411	688	689	887	893
SDXT2	A	Y	N	D	T	L	M	L
S1	A®R	-	-	D®N	T®I	-	-	-
S2	A®G	-	N®D	D®N	T®I	L®P	M®V	-
S3	-	Y®N	-	D®N	T®I	-	-	-
S4	A®G	-	-	D®N	T®I	-	-	L®P

**Figure-4:**
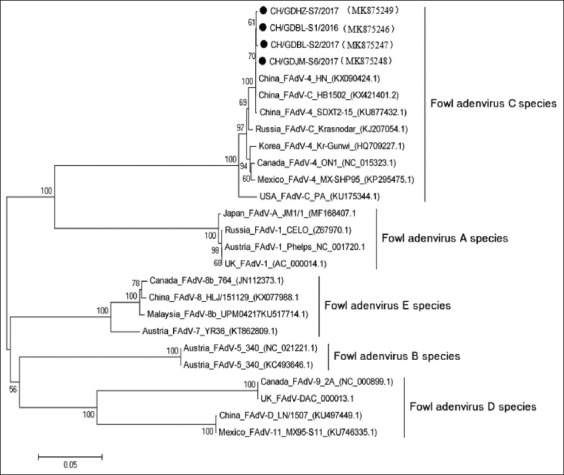
Phylogenetic analysis of nucleotide sequences of the *hexon* genes.

The nucleotide sequences of the amplified *hexon* genes and their deduced AA sequences were 99.8-99.9% and 99.5-99.8% identical to one another, respectively. Similarly, the nucleotide and deduced AA sequences revealed 97-100% (96.4-99.9%) identity with FAdV-4 gene sequences (FAdV-C strains) retrieved from the National Center for Biotechnology Information GenBank database; by contrast, their nucleotide and AA sequences were 76.0-76.3% (80.4-81%), 73.2-73.3% (76.8-77.1%), 69.6-71.7% (64.2-77.7%), and 74.8-75.1% (77.6-78.2%) identical to those of the FAdV-A, FAdV-B, FAdV-D, and FAdV-E strains, respectively. Phylogenetic analysis, based on the *hexon* gene sequence, revealed that the new isolates were most closely related to the reference FAdV-C strains; FAdV-A, FAdV-B, FAdV-D, and FAdV-E strains clustered in different branches of the phylogenetic tree (Figure-4).

## Discussion

HPS was first described in 1987 in broiler chickens in Karachi, Pakistan. Typical macroscopic HPS lesions include a gelatinous substance accumulating in the pericardial sac, accompanied by an enlarged liver [11]; the viscera of infected animals undergo varying degrees of bleeding [12]. The findings in our current study are consistent with these disease descriptions [13,14]. FAdVs are identified routinely in chickens on poultry farms throughout the world. Before 2014, circulating strains of FAdV-4 were recorded in China, although no outbreaks of severe HPS were reported [15]. Since July 2015, HPS outbreaks that are associated with high rates of morbidity and mortality [14] have occurred. HPS has been reported throughout world, including in India, Canada, Hungary, Korea, Japan, and Poland [7]. The emergence of hepatitis-HPS in China has been attributed to FAdV species C serotype-4 (FAdV-4) [16]; FAdVs remain in circulation among broilers, domestic chickens, and egg-layers, resulting in serious economic losses in the poultry industry [16].

In this study, four novel FAdV-4 strains were isolated from tissues from diseased and deceased chickens suspected of having HPS (also known as Angara disease). We subjected these isolates to experimental validation and conventional PCR assays. Among our findings, the gross pathology was consistent with that observed in earlier reports. All four of the pathogenic FAdV strains isolated in this study were genetically related to serotype-4 based on the sequence analysis of the amplified *hexon* gene; this is currently the dominant serotype circulating in Guangdong Province, China [16]. Our results suggest that continued surveillance of FAdVs is needed to monitor the spread of this virus and virus-associated HPS.

The novel FAdV-4 isolates identified in this study are highly homologous to one another, with 99.8-99.9% nucleotide sequence homology within the amplified *hexon* gene. The FAdV-4 isolates were also closely related to both domestic and foreign FAdV-C strains, displaying the 97-100% nucleotide sequence identity. The novel FAdV-4 isolates were more distantly related to FAdV-A, FAdV-B, FAdV-D, and FAdV-E strains, with nucleotide identities determined at 76.0-76.3, 73.2-73.3, 69.6-71.7, and 74.8-75.1, respectively.

A phylogenetic tree, based on the sequences of the *hexon* genes, generated five distinct clusters. As anticipated, the novel FAdV-4 strains clustered with those identified as FAdV-C; the *hexon* genes from FAdV-A, FAdV-B, FAdV-D, and FAdV-E strains generate distinct clusters, similar to results determined in the previous studies [17].

The hexon protein is the main surface antigen of avian adenovirus; its epitopes are comparatively conserved, and there is a high degree of sequence conservation among strains. We identified only nine AA divergence sites in our comparison of the new isolates to the GenBank reference sequence. Further research will be needed to determine the relationship between nucleotide sequence variation, virulence, and outbreak frequency. These results highlight the conservation of the hexon protein as a target antigen for vaccine development.

## Conclusion

Infections with FAdV-4 have had an enormous impact on the poultry industry. Here, we explored the molecular epidemiology and characteristics of an outbreak of HPS at poultry farms in Guangdong, China. The viruses, isolated from infected chickens, were identified as FAdV serotype-4, of the subgroup FAdV-C. Sequence analysis revealed a high degree of conservation among the new isolates and the reference strains for this serotype. Since our results were based on a limited number of samples, evaluation of a more extensive set of virus isolates will be needed to confirm these preliminary findings.

## Authors’ Contributions

Conceptualization, HS, SE, and LZ; formal analysis, FY, YS, DW, CS, LW, HW, LX, KM, GJ, ZX, and LZ; investigation, FY, YS, DW, CS, LW, HW, LX, KM, GJ, ZX, and LZ; methodology, FY and YS; supervision, HS; and writing-review and editing, HS, SE, and LZ;. All authors have read and agreed to the published version of the manuscript.
